# Adhesion Characteristics of an Asphalt Binder–Aggregate Interface Based on Molecular Dynamics

**DOI:** 10.3390/ma18050981

**Published:** 2025-02-23

**Authors:** Hao Xiang, Zhengxing Wang, Mingyang Deng, Silu Tan, Haoning Liang

**Affiliations:** 1 College of Civil Engineering and Architecture, Southwest University of Science and Technology, Mianyang 621010, China; 2 Key Laboratory of Engineering Materials of Ministry of Water Resources, China Institute of Water Resources and Hydropower Research, Beijing 100038, China

**Keywords:** asphalt binder, interface adhesion, molecular dynamics, contact angle, interfacial energy

## Abstract

To investigate the adhesion properties of asphalt binder–aggregate interfaces, contact angle tests were performed on base, aged, and SBS# asphalt with limestone and basalt aggregates. A molecular dynamics simulation model was established to analyze interfacial adhesion characteristics. The results indicate good consistency between the experimental and molecular dynamics simulation findings. SBS# asphalt exhibited superior surface free energy and adhesion properties compared with base asphalt, whereas aging reduced the adhesion performance. The interfacial energy between the asphalt and aggregates is closely related to their molecular compositions. When moisture penetrates the asphalt mixture, the interfacial energy between limestone and asphalt significantly increases, compared with that of basalt, with SBS# asphalt exhibiting stronger adhesion than base asphalt. The modifier enhanced the adhesion between the asphalt and aggregates, thereby providing resistance to moisture damage. The findings of this study possess referential value for the selection, modification, and performance optimization of asphalt pavement materials.

## 1. Introduction

A well-developed transportation infrastructure is fundamental for economic development. Owing to their advantages, such as driving comfort and ease of construction, asphalt pavements have become a major component of newly constructed highways. However, under the influence of traffic loads and environmental changes, asphalt pavements are susceptible to early stage distress, such as moisture damage, which significantly affects their service lives and comfort. These stresses are primarily caused by insufficient adhesion at the asphalt binder–aggregate interface [1]. Consequently, extensive research has been conducted on the adhesive properties of the asphalt–aggregate interfaces.

The electrostatic potential at the interface between asphalt molecules and aggregate oxides is the primary factor responsible for the adhesion between the asphalt and aggregates [2]. Aging alters the electrostatic interactions between the asphalt and aggregates [3]. There are differences in adhesion between different aggregates and asphalt, with a higher content of alkaline oxides favoring interfacial bonding [4,5]. Cala [6] proposed a predictive model for moisture damage in an asphalt–aggregate system based on the chemical composition of the aggregates. Meanwhile, the micro-roughness of the aggregate also has a significant impact on the adhesion rate of different aggregates [7,8]. For asphalt binder, the Saturates, Aromatics, Resins, and Asphaltene (SARA) components have the most significant impact on the interfacial bonding strength [5]. It can be seen that the properties of asphalt and aggregate have an important impact on interfacial adhesion.

Aging has an adverse effect on the interfacial interaction and bonding between asphalt binder and aggregate, ultimately affecting the performance of asphalt mixtures and pavement [9,10]. Natural conditions, such as light, oxygen, and water, also have a significant impact on the adhesion of asphalt pavements [11]. The asphalt–aggregate interface is particularly susceptible to moisture damage, leading to a reduction in the interfacial bonding strength [12]. Mihandoust [13] and Meng [14] confirmed that acidic environments accelerate the detachment of asphalt films, weaken intermolecular interactions, reduce adhesive energy, and lead to failure. The adhesion of an asphalt aggregate system can be measured based on the detachment of water from different materials [15]. However, results obtained from a single testing method should not rely solely on the final evaluation [16,17].

Owing to the limitations of laboratory experiments, molecular dynamics (MD) simulations have provided new insights into this field. Gong [18] employed MD simulations to study the effects of environmental factors on the adhesion characteristics of an asphalt–aggregate interface. Tang [19] and Xu [20] reviewed the application of molecular dynamics in asphalt–aggregate interfaces and the progress made in evaluating asphalt adhesion performance, focusing on model construction insights and interfacial performance assessment methods. MD simulations of the temperature and humidity characteristics at the asphalt–aggregate interface show that the simulation results are in good agreement with the results of micro-scale experiments [21,22].

Overall, although significant research has been conducted on the adhesion between asphalt and aggregates, most studies have focused on the adhesion between a single type of asphalt and an aggregate. Moreover, there has been a lack of validation between laboratory experiments and numerical simulations. Therefore, this study aims to combine laboratory experiments and molecular dynamics simulations to investigate the adhesion characteristics at the asphalt binder–aggregate interface to clarify the adhesion mechanisms between the asphalt binder and aggregates. These findings are expected to support the selection and modification of asphalt pavement materials and promote the application of molecular dynamics in the field of engineering materials.

## 2. Materials and Methods

### 2.1. Asphalt Binder and Aggregates

The experiment used 70# and SBS# asphalt sourced from Chongqing Zhixiang Paving Technology Engineering Co., Ltd. (Chongqing, China). The penetration values were 68 and 57 dmm at 25 °C, the softening points were 48.5 °C and 76 °C, and ductility values were >100 cm at 15 °C and 58 cm at 5 °C. The preparation process of SBS# asphalt is as follows: The 70# asphalt was heated to 160 °C, and then the Styrene-Butadiene-Styrene (SBS) modifier was added at a mass fraction of 5.5%. A high-speed shear disperser was used to shear at a rate of 6000 r/min for 30 min at about 180 °C to ensure the uniform dispersion of SBS modifier in the asphalt and the full swelling and dispersion of SBS modifier. A rotational thin-film oven test was employed to simulate asphalt aging at a heating temperature of 163 °C and heating time of 85 min. After aging, the residual penetration values for the 70# and SBS# asphalts were 47 mm and 45 mm, respectively.

The aggregates used were limestone and basalt, with a particle size distribution of 5–10 mm. Table 1 lists their basic properties determined in accordance with the Standard Test Methods of Aggregates for Highway Engineering (JTG E42-2005).

### 2.2. Contact Angle Test

The contact angle measurements were conducted using a Krüss DSA30 contact angle goniometer (KRUSS Company in Hamburg, Germany) at a temperature of 25 °C. The asphalt contact angle samples were prepared using the dipping method. For the aggregate contact angle tests, the aggregate surfaces were polished using sandpaper, washed several times with distilled water, and oven-dried until a constant weight was achieved. Distilled water and formamide were used as testing liquids. The surface free energy is composed of two components: the dispersive component ($\gamma_{L}^{D}$) and polar component ($\gamma_{L}^{P}$), as shown in Table 2.

According to the classic Young–Dupre equation, the following equation can be obtained:

(1) $$\gamma_{L}(1+\cos\theta )=2(\sqrt{\gamma_{L}^{D}\gamma_{S}^{D}}+\sqrt{\gamma_{L}^{P}\gamma_{S}^{P}})$$

where $\gamma_{L}$ is the total surface free energy of the object and θ is the contact angle between the liquid and solid.

Combined with the surface free energy theory, the adhesion work between the asphalt and aggregate can be obtained [23]:

(2) $$W_{ad}=2(\sqrt{\gamma_{a}^{D}\gamma_{h}^{D}}+\sqrt{\gamma_{a}^{P}\gamma_{h}^{P}})$$

where $W_{ad}$ is the adhesion work between the asphalt and aggregate and $\gamma_{a}^{D}$, $\gamma_{h}^{D}$, $\gamma_{a}^{P}$, and $\gamma_{h}^{P}$ are the dispersion and polar components of the asphalt and aggregate, respectively.

When water penetrates the asphalt mixture, it flushes the asphalt film wrapped around the aggregate surface [24], which causes the water to replace the asphalt film on the aggregate surface. This energy is the stripping work, and its calculation method is shown in (3):

(3) $$W_{de}=2(\sqrt{\gamma_{w}^{D}\gamma_{h}^{D}}+\sqrt{\gamma_{w}^{P}\gamma_{h}^{P}}+\sqrt{\gamma_{w}^{D}\gamma_{a}^{D}}+\sqrt{\gamma_{w}^{P}\gamma_{a}^{P}}-\sqrt{\gamma_{a}^{D}\gamma_{h}^{D}}-\sqrt{\gamma_{a}^{P}\gamma_{h}^{P}})$$

where $W_{de}$ is the stripping work between the asphalt and aggregate and $\gamma_{w}^{D}$ and $\gamma_{w}^{P}$ are the dispersion and polar components of water, respectively.

### 2.3. Molecular Dynamics Simulation

Asphalt is a complex mixture of chemicals. In this study, a four-component model was used to construct a base asphalt molecular model [25]. Oxygen atoms were introduced at the easily oxidized sites of the asphaltene, oil, and aromatic fractions, leading to the formation of carbonyl and sulfoxide groups, thereby simulating the asphalt aging process.

**Figure 1:**
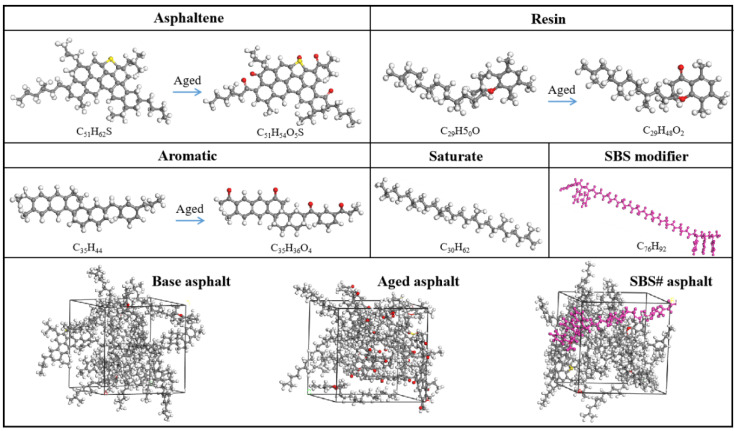
Molecular models of asphalt components and modifiers.

An asphalt molecular model was established based on this model. Figure 1 shows the molecular model for each component. Asphaltene, resin, aromatics, and saturation accounted for 11.1%, 25.8%, 43.4%, and 19.7% in the base asphalt; 11.5%, 31.5%, 38.3%, and 18.7% in the aged asphalt; and 10.5%, 24.3%, 39.6%, and 19.2% in the SBS# asphalt model, respectively. The mass fraction of the modifier was 6.4%. The molecular structure models of the base asphalt, aged asphalt, and SBS# asphalt were obtained via calculations in an amorphous cell module. The force field was calculated using Condensed-phase Optimized Molecular Potentials for Atomistic Simulation Studies (COMPASS), and the Quality level was fine. The lattice sizes of the three asphalt molecules were 22.0 Å × 22.0 Å × 22.0 Å, 22.4 Å × 22.4 Å × 22.4 Å, and 23.1 Å × 23.1 Å × 23.1Å, respectively, and the angles in the three directions were all 90°. The two asphalt molecular structure models were optimized using the Geometry Optimization tool, and 20 annealing treatments were performed using the Forcite module and NVT ensemble. The models were optimized after each annealing cycle. After 20 annealing cycles, the model with the lowest system energy and most stable structure was selected.

**Figure 2:**
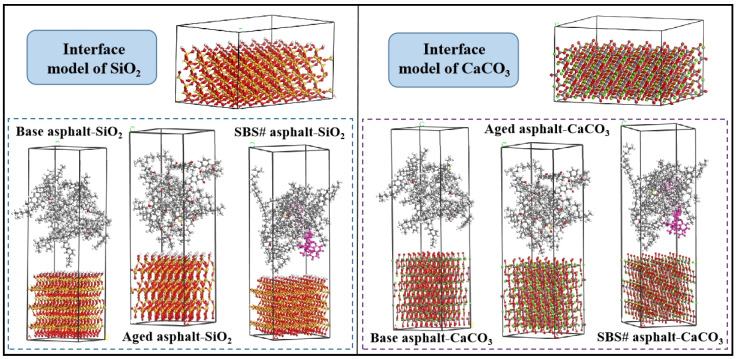
Aggregate molecular model.

The CaCO_3_ and SiO_2_ supercell models were constructed to represent limestone and basalt, respectively [26,27]. The model first processed the mineral crystals and then expanded the supercell to a size close to that of asphalt molecules. Finally, a vacuum layer was added to the mineral surface to transform the two-dimensional periodic structure into a three-dimensional periodic structure. Figure 2 shows the three-dimensional aggregate molecular model. The asphalt–aggregate interface was established using the Build Layer function. The first and second layers in the model were aggregate and asphalt, respectively. To avoid this periodic effect, a vacuum layer of 100 × 10^−10^ m was added to the asphalt layer, and the interface model was optimized geometrically and dynamically.

## 3. Results

### 3.1. Results of Contact Angle Test

**Figure 3:**
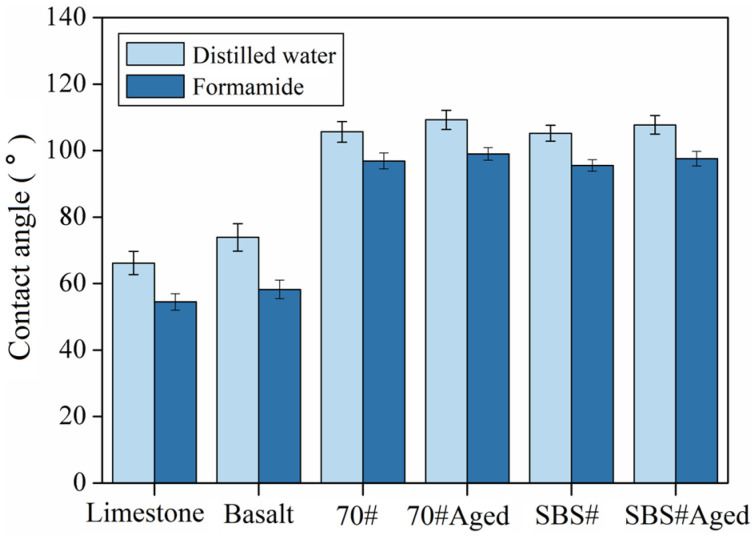
Contact angle test results of asphalt and aggregate.

Figure 3 shows the contact angle test results for the asphalt, aggregate, and liquid. The contact angle between basalt and the liquid is greater than that between limestone, indicating that limestone is more easily wetted by liquids. When not aged, the contact angle between SBS# asphalt and the liquid was smaller than that between SBS# asphalt and 70# asphalt. However, the values were similar, with a difference of less than 1.5%. After aging, the contact angles of asphalt #70 and #SBS increased significantly. When in contact with distilled water, the contact angles increased by 3.4% and 2.4%, respectively. When in contact with formamide, the contact angles increased by 2.2% and 2.1%, respectively, indicating that aging increased the hydrophobic properties of asphalt and that 70# asphalt was more sensitive to thermal oxidative aging.

### 3.2. Results of Surface Free Energy

**Figure 4:**
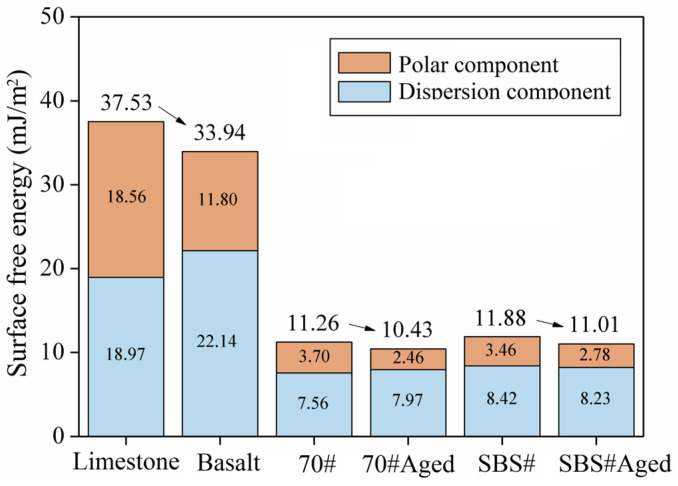
Surface free energy parameters of asphalt and aggregate.

Figure 4 shows the calculation results for the surface free energy parameters as well as the asphalt and aggregate components based on the contact angle test results. Compared with 70# asphalt, the surface free energy of SBS# asphalt increased by 5.5%. The surface SBS modifier increased the surface free energy of asphalt because the SBS modifier forms a stable spatial network structure with the base asphalt. Simultaneously, the SBS modifier absorbs some aromatic and saturated components and converts them into colloids and asphaltene with stronger polarity, thereby increasing the intermolecular forces. In contrast, the surface free energy of asphalt #70 decreases by 7.4% after aging, indicating that the effect of aging is also significant. The surface free energy of limestone was greater than that of basalt, mainly because of the difference between the polar components. Regardless of whether asphalt or aggregate is used, the gap between the dispersion components is smaller than that between the polar components, which ultimately shows a different total surface free energy.

### 3.3. Results of Adhesion and Peeling

**Figure 5:**
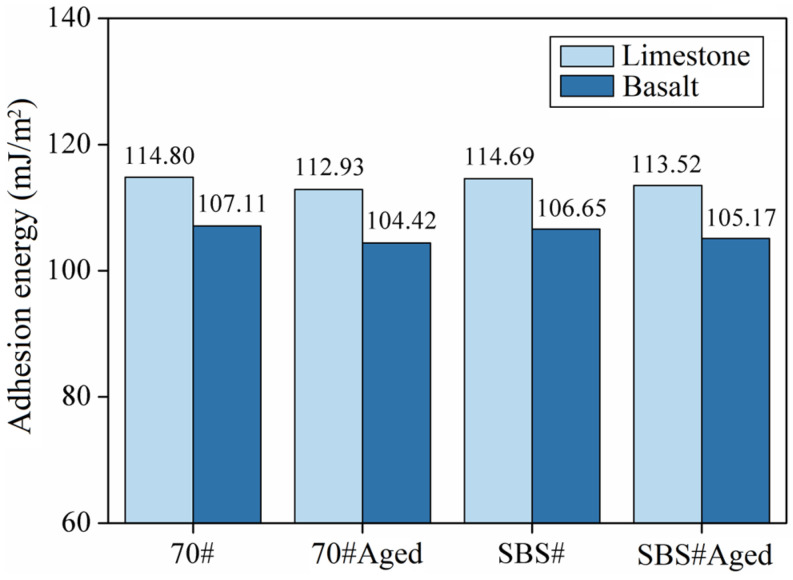
Adhesion energy between asphalt and aggregate.

**Figure 6:**
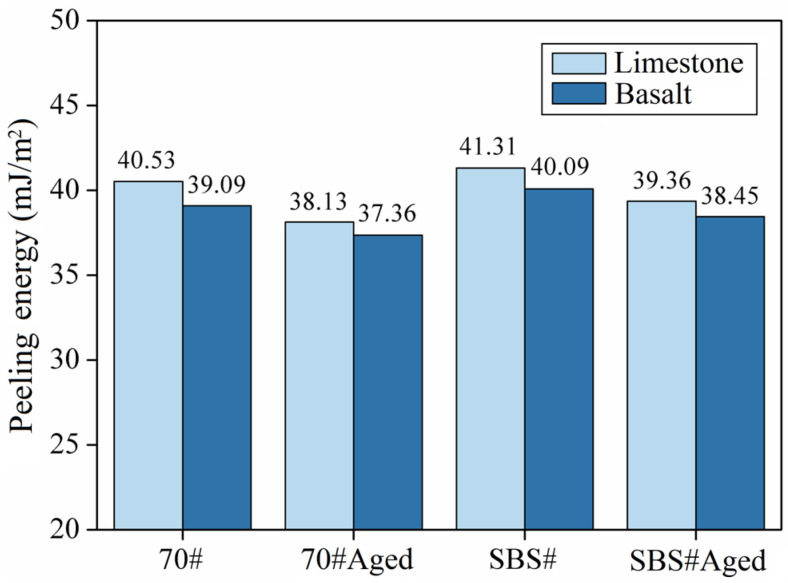
Peeling energy of asphalt and aggregate.

The adhesion and peeling work of the asphalt and aggregate under water conditions were calculated according to Formulas (1)–(3). Figure 5 and Figure 6 show the results. In this study, the adhesion work of asphalt to limestone was significantly greater than that of basalt, owing to the difference in the chemical composition of the aggregates. The main component of limestone is CaCO_3_, whereas that of basalt is SiO_2_. Alkaline aggregates are conducive to asphalt adhesion, whereas SiO_2_ has an adverse effect. For the same type of aggregate, the adhesion work generally followed the order 70# > SBS# > SBS#Aged > 70#Aged, but the values for 70# asphalt and SBS# asphalt were close. In general, the variation in the adhesion work of the 70# asphalt after aging is larger, which indicates that the 70# asphalt is more easily affected by aging.

Figure 6 shows the stripping work of the asphalt and aggregate. Compared with limestone, the stripping work between asphalt and basalt was smaller. Under the same aggregate, SBS# asphalt and aggregate have the largest stripping work, and 70#Aged asphalt has the smallest stripping work. A reduction in the stripping work means that the force required for water to separate the asphalt attached to the aggregate is reduced, and the asphalt is more susceptible to water damage. A comprehensive comparison shows that the stripping work between asphalt and aggregate ranges from large to small: SBS# > 70# > SBS#Aged > 70#Aged, indicating that the addition of modifiers is beneficial for resisting water damage between asphalt and aggregate; however, the modifier has a small improvement in asphalt adhesion.

### 3.4. Results of Model Verification

**Figure 7:**
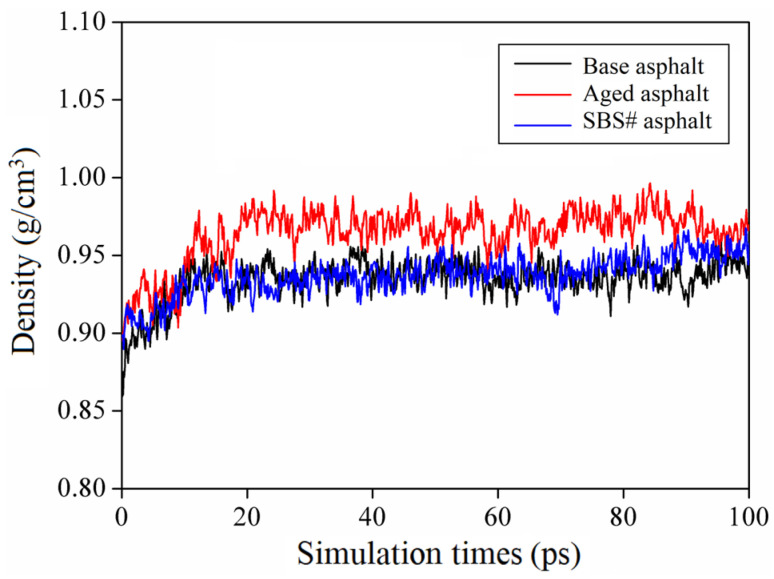
Relationship between density and temperature.

Density was used to verify the validity of the asphalt model and ensure its correctness. The density calculation used the dynamics mode in the Forcite module, selected the COPASS force field and a fixed pressure (0.0001 GPa), and calculated the density of the asphalt molecular model at 298 K. The density calculation time was 100 ps, and Figure 7 shows the density of the asphalt model in the range of 0–100 ps. According to the results in Figure 7, the density change rules of the three asphalt models are consistent and manifest as small fluctuations over time. The average densities of the base, aged, and modified asphalts in the stable stage after 20 ps were 0.938, 0.962, and 0.955 g/cm^3^, respectively. The aging effect and addition of modifiers increased the density of the asphalt, and the numerical value and change rule were consistent with the actual situation.

**Figure 8:**
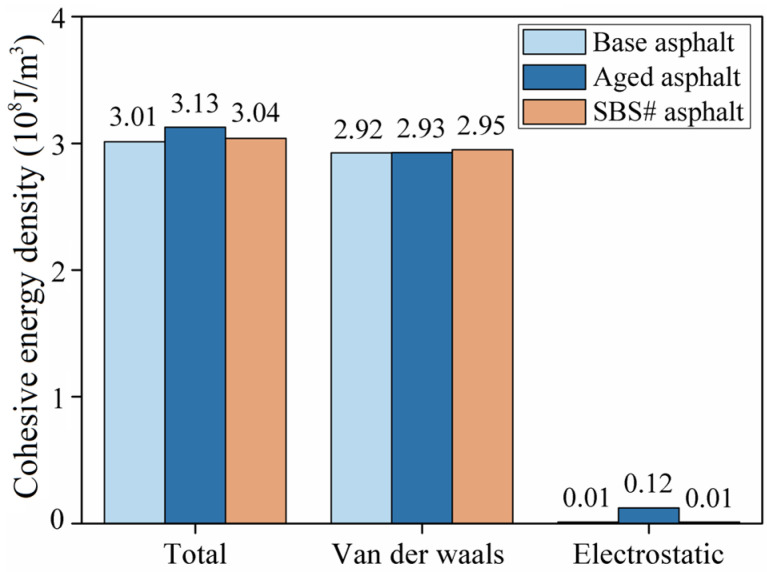
Simulation results of the cohesive energy density.

The cohesive energy density refers to the energy required per unit volume for an object to vaporize and overcome intermolecular forces. Moreover, the cohesive energy density is a physical quantity used to evaluate the magnitude of intermolecular forces, mainly reflects the interactions between groups, and is an indicator of the strength of intermolecular forces. Figure 8 shows the cohesive energy density of each asphalt model at 298 K. The total cohesive energy density of the asphalt after aging or modification increased slightly, indicating that the internal interaction forces of the asphalt molecules were enhanced. The cohesive energy density of the asphalt material in the model was mainly determined by the van der Waals forces. The cohesive energy density provided by the electrostatic forces was negligible [28,29].

### 3.5. Results of Interface Energy

The total energy ($E_{t}$), asphalt energy ($E_{a}$), and aggregate energy ($E_{s}$) of the asphalt and aggregate models were obtained via molecular dynamics calculations. The contact area between the two materials is (A), and the interfacial energy can be calculated according to Equation (4) [19]:

(4) $$E_{\mathrm{interfacial}\;\mathrm{energy}}=(E_{total}-E_{asphalt}-E_{aggregate})/A$$

where $E_{\mathrm{interfacial}\;\mathrm{energy}}$ is the interfacial energy between the asphalt and aggregate, $E_{total}$ is the total interfacial potential energy, $E_{asphalt}$ and $E_{aggregate}$ are the potential energies of the asphalt aggregate, and A is the interfacial area.

**Figure 9:**
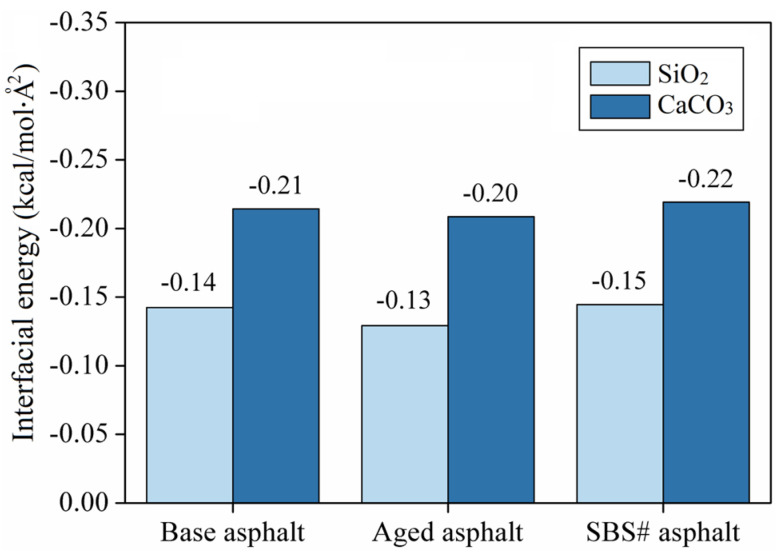
Interfacial energy of the asphalt–aggregate model.

Figure 9 shows the calculation results for the interface energy of the asphalt–aggregate model. In this study, the aggregate layer was fixed during modeling. Therefore, the total energy obtained when only the aggregate layer was retained was zero. The adhesion of the SiO_2_ and CaCO_3_ aggregates to asphalt was significantly different. The adhesion of the CaCO_3_ aggregate model to asphalt was better than that of SiO_2_. The CaCO_3_ aggregate model represents alkaline aggregate limestone, whereas the SiO_2_ aggregate model represents acidic aggregate. Basalt is an alkaline aggregate with low SiO_2_ content. The results showed that alkaline aggregates exhibit better adhesion to asphalt because the interface between the SiO_2_ aggregate model and asphalt mainly relies on intermolecular van der Waals forces, whereas the interface between the CaCO_3_ aggregate model and asphalt is mainly based on electrostatic forces. Therefore, the adhesion work of the SiO_2_ aggregate to asphalt was less than that of the CaCO_3_ aggregate. Owing to the different structural compositions of the asphalt, the interfacial energy was improved after adding the modifier and reduced after aging, which is consistent with the conclusions of the indoor test.

### 3.6. Results of Energy Ratio

The hydrophobicity of asphalt and hydrophilicity of aggregates can easily cause free water in the asphalt–aggregate interface to peel off the asphalt film on the aggregate surface and, thereby, affect the bonding between the asphalt and aggregate [30]. Therefore, when calculating the peeling work, this study added 98 water molecules to the middle of the asphalt and aggregate model to simulate the invasion of water into the asphalt–aggregate interface, thus, forming an “asphalt–water–aggregate” model. The adhesion performance between the asphalt and aggregate was further analyzed based on the damage caused by water to the asphalt–aggregate interface. Equations (5) and (6) were used to calculate the energy ratio ($E_{R}$) to quantitatively evaluate the water damage resistance of asphalt mixtures [12]:

(5) $$W_{debonding}=(E_{\mathrm{int}er\_aw}+E_{\mathrm{int}er\_agw}-E_{\mathrm{int}er\_aag})/A$$

(6) $$E_{R}=W_{adhesion}/W_{debonding}$$

where $E_{R}$ is the energy ratio; $W_{debonding}$ is the stripping work; $E_{\mathrm{int}er\_aw}$, $E_{\mathrm{int}er\_agw}$, and $E_{\mathrm{int}er\_aag}$ represent the interfacial energy between the asphalt and water molecules, aggregate and water molecules, and asphalt and aggregate, respectively.

**Figure 10:**
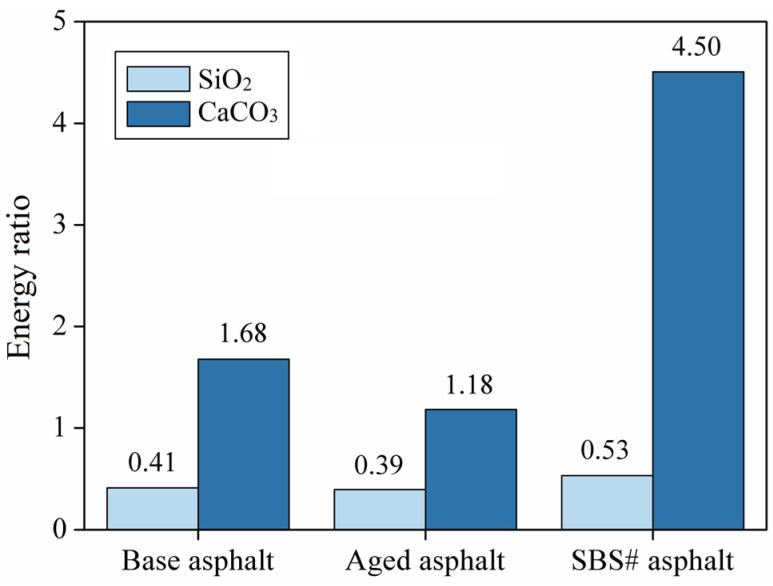
Asphalt–aggregate model interface energy.

Figure 10 shows the energy ratio between the asphalt and aggregate, calculated based on the results of the molecular dynamics model. The results show that the energy ratio between the SBS# asphalt and aggregate is the largest, followed by base asphalt, with aged asphalt being the smallest. These results indicate that SBS# asphalt has the best adhesion to the aggregate and the best resistance to water damage, whereas aged asphalt has the worst. The energy ratio of the CaCO_3_ aggregates is higher than those of the SiO_2_ aggregates. For example, the energy ratio of the base asphalt to the CaCO_3_ aggregate was four times that to the SiO_2_ aggregate. The differences between aggregates have a more significant impact on the asphalt–aggregate adhesion properties than the differences between asphalt types.

### 3.7. Results of Mean Square Displacement

Considering that asphalt molecules continuously move at the aggregate interface, the MD software (Materials Studio 2020) defines the position difference of the motion vector of the particle at the initial moment and calculation moment as the mean square displacement (MSD). The MSD can effectively reflect the motion state of the molecule [31,32]. Equation (7) represents the MSD calculation method:

(7) $$MSD(t)={\langle r_{i}(t)-r_{i}(0)\rangle }^{2}$$

where MSD(t) is the mean square displacement of the particle at the calculation time and $r_{i}(0)$ and $r_{i}(t)$ are the motion vector positions of the particle at the initial time and calculation time, respectively.

**Figure 11:**
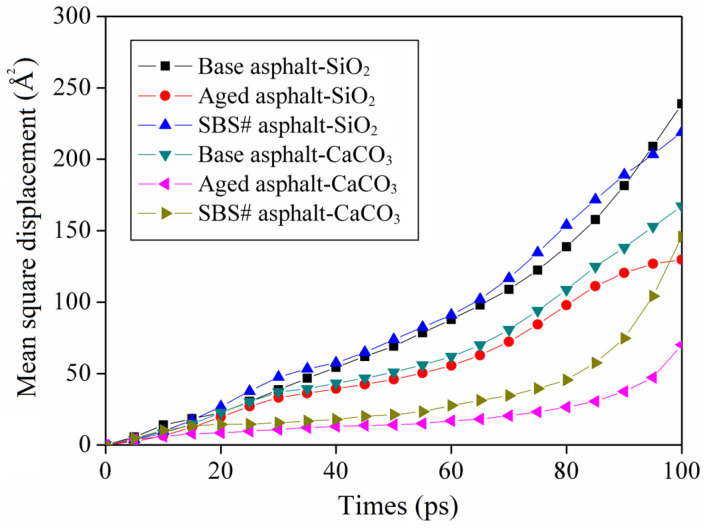
Mean square displacement of asphalt at the aggregate interface.

Figure 11 shows the mean square displacement of the asphalt model at different aggregate interfaces at 298 K. The mean square displacement of the asphalt atoms gradually increased with time. The curve can be divided into two stages based on the growth rate of the MSD. The first stage was a slow growth stage that occurred 50 ps before the start of healing. The mean square displacement of the asphalt model shows a small slope increase and steady increase during this stage. A rapid growth stage occurred 50 ps after healing. This stage indicates that the diffusion system gradually escaped the constraints of the intermolecular forces.

After thermal oxidation, the diffusion coefficient of the asphalt is lower than that of the original asphalt. The mean square displacement of the base asphalt and CaCO_3_ aggregate interface before and after aging decreases from 238.7 Å^2^ to 70.1 Å^2^, constituting a decrease of more than 70%. It is more difficult for asphalt molecules to diffuse at the interface of CaCO_3_ aggregates than at the interface of SiO_2_ aggregates, with a diffusion coefficient of approximately 50–70% times. This may be because asphalt undergoes chemical adsorption on the surface of CaCO_3_, which restricts the movement of asphalt molecules at the interface and thereby reduces diffusion. The SiO_2_ aggregate is acidic and more physically adsorbed. Therefore, the diffusion coefficient of the bio-asphalt molecules at its interface is larger, has better mobility, and breaks free from the constraints of the aggregate interface more easily, which causes damage and reduces adhesion.

## 4. Conclusions

In this study, we calculated the asphalt–aggregate adhesion energy and peeling work using contact angle tests and established an asphalt–aggregate interface adhesion model using MD software. After the model was verified as correct, the asphalt–aggregate adhesion was calculated. The evaluation parameters and results of the combination of the indoor tests and numerical simulations are summarized as follows:

(1) The indoor test results were consistent with the conclusions of the MD numerical simulations. The adhesion of limestone to asphalt was significantly better than that of basalt. The alkaline aggregate forms a strong interface with asphalt through electrostatic forces, whereas the acidic aggregate mainly relies on van der Waals forces, resulting in a weak adhesion performance. In addition, the SBS modifier can improve the adhesion between asphalt and aggregates; however, the improvement in resistance to water damage is limited;
(2) The calculation results of the interfacial energy and energy ratio show that SBS-modified asphalt has the best adhesion with aggregates and a strong resistance to water damage. In contrast, both the energy ratio and interfacial energy of the aged asphalt decreased significantly, indicating that aging significantly reduced the adhesion of asphalt to aggregates and its resistance to water damage;
(3) Thermal oxidative aging caused the diffusion coefficient of asphalt at the interface of the CaCO_3_ aggregate to decrease significantly, with a decrease of more than 70%, whereas the diffusion coefficient at the interface of the SiO_2_ aggregate was higher, indicating that the different behaviors of asphalt on the surfaces of different types of aggregates affect the final physical properties and significantly impact durability.

## Figures and Tables

**Table 1:** Technical Indexes of Aggregates.

Type	Apparent Density (g/cm^3^)	Water Absorption (%)	Crushing Value (%)	Los Angeles Abrasion Loss (%)
Limestone	2.715	0.67	17.1	14.8
Basalt	2.873	0.52	13.2	12.7

**Table 2:** Surface Free Energy and its Components of Reference Liquids.

Type	Total Surface Free Energy (mJ/m^2^)	Dispersive Component (mJ/m^2^)	Polar Component (mJ/m^2^)
Distilled Water	72.8	21.8	51.0
Formamide	58.2	39.5	18.7

## Data Availability

The original contributions presented in this study are included in the article. Further inquiries can be directed to the corresponding author.
